# Use of High-Throughput Sequencing and Two RNA Input Methods to Identify Viruses Infecting Tomato Crops

**DOI:** 10.3390/microorganisms9051043

**Published:** 2021-05-12

**Authors:** Ayoub Maachi, Covadonga Torre, Raquel N. Sempere, Yolanda Hernando, Miguel A. Aranda, Livia Donaire

**Affiliations:** 1 Abiopep S.L., Parque Científico de Murcia, 30100 Murcia, Spain; a.maachi@abiopep.com (A.M.); ctorre@abiopep.com (C.T.); rnsempere@abiopep.com (R.N.S.); yh.saiz@abiopep.com (Y.H.); 2 Department of Stress Biology and Plant Pathology, Centro de Edafología y Biología Aplicada del Segura (CEBAS)-CSIC, 30100 Murcia, Spain; m.aranda@cebas.csic.es

**Keywords:** high-throughput sequencing (HTS), tomato, virus, dsRNA, total RNA, OLV1, LRNV, ToFBV

## Abstract

We used high-throughput sequencing to identify viruses on tomato samples showing virus-like symptoms. Samples were collected from crops in the Iberian Peninsula. Either total RNA or double-stranded RNA (dsRNA) were used as starting material to build the cDNA libraries. In total, seven virus species were identified, with pepino mosaic virus being the most abundant one. The dsRNA input provided better coverage and read depth but missed one virus species compared with the total RNA input. By performing in silico analyses, we determined a minimum sequencing depth per sample of 0.2 and 1.5 million reads for dsRNA and rRNA-depleted total RNA inputs, respectively, to detect even the less abundant viruses. Primers and TaqMan probes targeting conserved regions in the viral genomes were designed and/or used for virus detection; all viruses were detected by qRT-PCR/RT-PCR in individual samples, with all except one sample showing mixed infections. Three virus species (*Olive latent virus 1*, *Lettuce ring necrosis virus* and *Tomato fruit blotch virus*) are herein reported for the first time in tomato crops in Spain.

## 1. Introduction

Tomato (*Solanum Lycopersicum* L.) is one of the most important vegetable crops. The worldwide production of tomato in 2019 was more than 182 million tons (FAOSTAT, 2019; http://www.fao.org/faostat/; accessed on September 2020). Spain is one of the world’s leading producers of tomato plants for fresh consumption, second in the European Union after Italy (Eurostat 2019; https://ec.europa.eu/eurostat/371; accessed on September 2020). Tomato cultivation in Spain is very intensive, with significant acreage devoted to greenhouse production. The major threats to tomato intensive cultivation are viral diseases, which are responsible for significant yield and fruit quality losses, causing important economic damage [1]. The main viruses frequently reported to affect tomato crops include tomato yellow leaf curl virus (TYLCV), pepino mosaic virus (PepMV), tomato spotted wilt virus (TSWV) and tomato chlorosis virus (ToCV), with all four of them having been reported in the Iberian Peninsula (EPPO). Nevertheless, emerging viruses, i.e., those recently reported and the incidence or geographic range of which increase rapidly [2], often cause the most important problems. A recent example of an emerging virus infecting tomato crops in the Iberian Peninsula is tomato brown rugose fruit virus (ToBRFV) (EPPO). The availability of sensitive and reliable virus discovery and detection techniques is crucial for diagnosing and controlling viral diseases, as well as anticipating problems that are potentially caused by major, emerging, or new viruses.

High-throughput sequencing (HTS) technologies enable, in a relatively short period of time, the characterization of plant viromes, allowing both the detection of known viruses and the discovery of novel ones [3]. These technologies have been successfully used with several crop species, including tomato plants [4,5,6,7,8,9]. The application of HTS to samples from tomato crops in China revealed the presence of 22 viruses, of which five of them had not been reported previously to infect plants of this species [8]. Another study, comparing the diversity of viral populations between tomato plants and neighboring *Solanum nigrum* plants using HTS, showed a large variability in virome richness, but with little overlapping of the viruses found in both species [9]. In addition to its detection potential, different works have shown that HTS can increase the resolution of virus population genetics and evolution studies and also allows the determination of the complete or near-complete genomes of novel viruses without any prior knowledge [3,10]. To date, different starting materials have been used for metagenomic studies, including double-stranded RNA (dsRNA), total RNA depleted of ribosomal RNA (rRNA-depleted total RNA), virion associated nucleic acids (VANA) and small RNAs (sRNAs). The comparison between methods using different starting materials has shown differences in the spectrum of viruses or viroids that can be detected. Previous works have shown that the outcome of rRNA-depleted total RNA-based methods tends to be virus-dependent; sRNA sequencing is better than rRNA-depleted total RNA for the detection of viroids [10,11] and both dsRNA and VANA allowed for the enrichment of virus sequences in the samples [12].

Here, we used the rRNA-depleted total RNA and dsRNA approaches to identify the viruses present in tomato samples from plants exhibiting virus-like symptoms. We compared the virus diversity and mapping reads between two replicates from total RNA extractions. In addition, we adopted an in silico approach to determine the minimum sequencing depth needed to detect the less abundant viruses in our sample pools. We identified seven known virus species, three of which are reported for the first time in tomato plants in Spain: *Lettuce ring necrosis virus*, *Olive latent virus 1*, and *Tomato fruit blotch virus*.

## 2. Materials and Methods

### 2.1. Plant Material

Twenty samples of tomato leaves exhibiting symptoms suggesting viral infection were collected during the 2015–2020 period from different locations in Spain and Portugal (Table 1). A portion of leaf tissue from each sample was placed in 1.5-mL tubes, frozen in liquid nitrogen, and stored at −80 °C until RNA extraction.

### 2.2. Nucleic Acid Extraction, Library Construction and Sequencing

For the total RNA extraction, 100 mg of leaf tissue from each individual sample was ground until attaining a fine powder with a mortar and pestle in the presence of liquid nitrogen. Total RNA was extracted using TRI Reagent (Sigma-Aldrich, St. Louis, MO, USA) following the manufacturer’s protocol. For the dsRNA extraction, 100 mg of leaf tissue from each sample was pooled and ground. dsRNA was purified using the protocol from Valverde et al. [13] with Whatman CF-11 cellulose powder (GE Healthcare Life Science Corp., Piscataway, NJ, USA). Both preparations were subjected to RNase-free DNase I treatment (New England Biolabs, Ipswich, MA, USA), following the manufacturer’s protocol, to remove traces of DNA, and the dsRNA preparation was also treated with RNase A (Machery-Nagel, Duren, Germany), following the protocol described in [14], to remove single-stranded RNA traces. After these treatments, the preparations were cleaned up by phenol/chloroform extraction [14] and their integrity was confirmed using gel electrophoresis. The quantity of the total RNA was assessed using a Nanodrop^TM^ One Microvolume UV-Vis Spectrophotometer (Thermo Fisher Scientific, Waltham, MA, USA) and a Qubit^TM^ 3.0 Fluorometer (Thermo Fisher Scientific), and individual samples were normalized to a final concentration of 20 ng/µL. Two identical pools denoted Tom1 and Tom2 were prepared by adding 7.5 μL from each total RNA sample to obtain a final amount of 3 μg of total RNA in 150 μL of sterile MiliQ water. The dsRNA sample (denoted as TomDS) had a final volume of 50 μL in sterile MiliQ water. The samples were sent to Macrogen (Seoul, Korea) for library preparation and sequencing. The quality and quantity of the RNA in the three samples were further analyzed using a 2100 Bioanalyzer (Agilent Technologies, Palo Alto, CA, USA). For the three samples, cDNA libraries were synthesized using a TrueSeq Stranded Total RNA sample preparation kit (Illumina, San Diego, CA, USA) with ribosomal depletion using a Ribo-Zero plant kit (Illumina). Sequencing was performed with the Illumina NovaSeq 6000 platform to obtain 150 bp paired-end reads.

### 2.3. Bioinformatic Analysis

**Figure 1:**
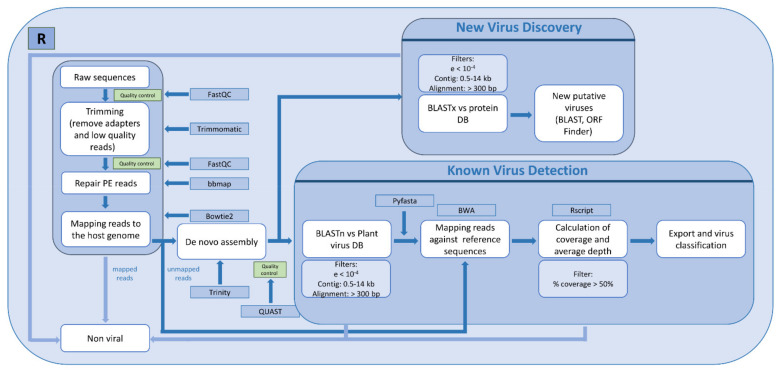
Bioinformatic workflow for the detection of known viruses and for novel virus discovery. Schematic representation of the bioinformatics pipeline followed in this work implemented in the R language. Specific programs (blue rectangles) used for each step (white rectangles) are indicated; applied filters are framed in light blue rectangles.

Raw reads were analyzed using the custom bioinformatic pipeline implemented in the R language, as described in Figure 1. Paired-end reads in fastq format served as the input. The quality of the raw reads was screened using the FastQC program (http://www.bioinformatics.babraham.ac.uk/projects/fastqc; accessed on May 2020). Adapters and low-quality reads (Phred < 30) were trimmed from each data set using Trimmomatic v0.39 [15]. After this step, paired-end reads were repaired using BBMap [16]. Host reads were filtered out by aligning reads to the host genome (Tomato genome version SL2.4) using Bowtie2 [17]. Unmapped reads were subjected to de novo assembly using Trinity v2.10 [18]. For virus detection, contigs were aligned against a custom plant virus nucleotide database using BLASTn [19]. To build the virus database, viral sequences fitting the criteria of host: Viridiplantae and sequence length: 800–23,000 nt were downloaded from NCBI Virus (https://www.ncbi.nlm.nih.gov/labs/virus/vssi/#/; accessed on May 2020). The database was built using makeblastdb, and low-complexity sequences were filtered out with dustmasker [20]. Sequences sharing 98% at both nucleotide (nt) and amino acid (aa) levels were collapsed using cd-hit [21]. After BLASTn, viral hits were filtered using the following criteria: contig length between 0.5 to 14 kb, e-value lower than 10^−4^, and length of alignment between the query and the hit ≥ 300 nt. Pyfasta v0.5.2 (https://pypi.org/project/pyfasta/; accessed on May 2020) was used to retrieve the sequences of the reference viruses detected. Only one random accession for each virus was retrieved in the case that more than one accession for the same virus was found by BLASTn. These viral sequences were used as the reference to re-map the filtered reads using BWA with the mem algorithm [22]. From these alignments, virus genome coverage and average depth were calculated using SAMtools [23] and our own R script. To compare the percentage of identity of a given virus across the three datasets, a consensus sequence was generated using SAMtools and the Seqtk tool [24]. In cases where we could not obtain good consensus sequences, the longest contigs were used for these comparisons (Table S1). To determine the closest viral isolate, the consensus or the contig sequences of each virus were screened against its taxon using the NCBI database with BLASTn. The presence of divergent viral sequences was investigated by mapping contigs against a custom plant virus protein database using BLASTx [19], using the same filters as mentioned above. Although we did not find any novel viruses in this work, our pipeline included a step to analyze potential new viral species: ORF Finder (https://www.ncbi.nlm.nih.gov/orffinder/; accessed on May 2020) to predict ORFs of new putative viruses, and BLASTx against non-redundant proteins NCBI database to find the closest virus species. Subsets of reads used to determine the minimum number of reads needed to detect the viruses present in the datasets were obtained with Seqtk [24], using different seed values (100 and 120) in case two replicates were generated.

### 2.4. Conventional RT-PCR and qRT-PCR 

All viruses were detected in individual samples by qRT-PCR, except southern tomato virus (STV), which was detected by conventional RT-PCR using the primer pair described in Table S2, Expand Reverse Transcriptase (Roche, Basel, Switzerland) and NXT Taq PCR Kit (EURx, Gdańsk, Poland) according to the manufacturer’s instructions. Primers and probes for PepMV were published elsewhere [25]. For the other viruses, forward and reverse primers, together with TaqMan probes, were designed to target conserved regions of the CP gene, except for tomato fruit blotch virus (ToFBV) for which we used the RdRp gene, using the PrimerQuest Tool from IDT (https://eu.idtdna.com; acceded on September 2020). The specificity of all the primers and probes was confirmed in silico by a BLASTn search against the NCBI database. Primers’ and probes’ sequence information and the length of the amplicons are detailed in Table S2. For virus detection by qRT-PCR, the KAPA PROBE FAST One-Step qRT-PCR kit (Roche, Basel, SZ) was used. Each 10-μL reaction consisted of 5 μL of 2× Master Mix, 0.2 μL of forward/reverse primers (10 μM) and probe (10 μM), 0.2 μL of 50× RT-Mix, 0.2 μL of 50× ROX high, 2 μL of DEPC-treated water and 2 μL of RNA (20 ng/μL). The performance of the primers/probe pairs was determined by calculating the PCR amplification efficiency of the reaction from a standard curve of five 1:10 serial dilutions of the pooled total RNA sample (200 ng/μL) (Table S2).

## 3. Results

### 3.1. HTS Using Two Different RNA Inputs: Total RNA and dsRNA

**Figure 2:**
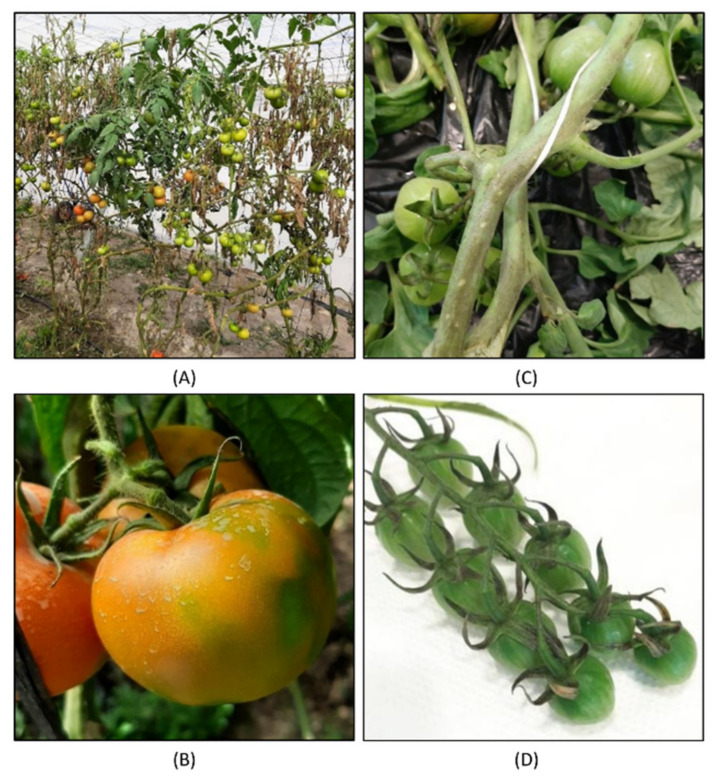
Tomato plants and fruits exhibiting virus-like symptoms. (**A**,**B**) correspond to the greenhouse where sample R19-07 was collected in Murcia. Tomato fruits exhibited fruit blotching and discoloration. (**C**,**D**) correspond to another greenhouse in Almería where sample R19-12 was collected, and tomato plants exhibited necrosis.

Twenty samples from leaves of tomato plants exhibiting virus-like symptoms were collected from 2015 to 2020 from different locations in the South of Spain and Portugal (Table 1). The sampled plants exhibited a wide range of disease symptoms suggestive of viral infection, i.e., vein clearing, leaf distortion, leaf curling, necrotic spots or mosaics on leaves (Table 1). Figure 2 shows representative examples of symptoms found in two different greenhouses, displaying fruit blotching, uneven ripening and necrosis. From these samples, we prepared two different RNA inputs. Total RNA was purified from individual samples and then pooled; dsRNA was extracted in a single preparation from an equivalent pool of samples. We sequenced two different libraries from the total RNA pool, representing two technical replicates (Tom1 and Tom2) and one from the dsRNA extraction (TomDS).

After sequencing the three libraries, we obtained 86,284,538, 84,739,174 and 64,540,826 reads for Tom1, Tom2 and TomDS, respectively (Table 2). The raw reads were analyzed following the pipeline described in Figure 1. These were trimmed and filtered to remove low-quality bases, and tomato-derived sequences were extracted by mapping against the tomato genome (Table 2). As expected, the percentage of reads mapping to the plant genome was much lower using dsRNA (25.7% of the clean reads) than using total RNA as the input (around 48% of the clean reads) (Table 2). Hence, a total of 44,121,432, 43,524,150 and 47,320,422 filtered reads corresponding to 51.9%, 52.1% and 74.3% of the clean reads were further used for virus identification in Tom1, Tom2 and TomDS, respectively (Table 2). Although the number of raw reads sequenced for the two total RNA samples was higher than for the dsRNA sample (around 80 M compared to around 60 M), the number of reads after the application of different quality filters was similar for the three libraries (Table 2).

### 3.2. Comparison of Viral Species Found in Two Technical Replicates Using Total RNA as the Input

In order to evaluate the reproducibility of the library construction and sequencing, the results from the two independent libraries, Tom1 and Tom2, were compared. The filtered reads were de novo assembled, and long contigs were mapped by BLASTn against our own plant virus database (Figure 1 and Table 2). After filtering the BLASTn results, we obtained a total of 63 and 51 contigs, with an average length of 2788 nt and 3076 nt, that mapped to viral sequences for Tom1 and Tom2, respectively (Table 2). To ensure high confidence in the detection of viruses, we set a minimum threshold of the assembled contig of 500 nt in length. In both replicates, we identified the same seven virus species (Table 3). No novel viruses were found by BLASTx using our pipeline.

To calculate the average sequencing depth and the genome coverages, the filtered reads were mapped against the reference sequences of the identified viruses. In the cases where multiple accessions were found for the same virus species, the accession of the reference sequence used to map the reads was randomly selected. Viral reads constituted 7.99% and 8.57% of the clean reads for Tom1 and Tom2, respectively (Table 2). The number of reads mapping to viral genomes varied from 390 (Tom1) and 364 (Tom2) reads, counted for olive latent virus 1 (OLV1), to 6,431,722 (Tom1) and 6,809,266 (Tom2), for PepMV (Table 3). The average sequencing depth varied from nine for OLV1 in either Tom1 and Tom2 to ~7690 for PepMV in either Tom1 and Tom2 (Table 3). The lowest percentage of coverage along the corresponding viral genome was found for OLV1 (97.97% in Tom1 and 89.68% in Tom2) and RNA1 from ToCV (96.92% in Tom1 and 94.16% in Tom2) (Table 3). For the other virus species, the percentage of coverage was higher than 98% (Table 3). The nucleotide sequence identity among the viruses found in the two replicates was higher than 98.9% (Table S1). Overall, our results indicate that reproducibility using total RNA after rRNA depletion is very high, as no significant differences were observed among the results obtained here for the two replicates.

### 3.3. Viral Species Found Using Total RNA or dsRNA as the Input

Procedures for sample preparation and RNA extraction for dsRNA and total RNA were obviously different, but the amount of plant material used for both methods was the same, allowing some comparisons. Since Tom1 and Tom2 are almost identical replicates, only the comparison between TomDS and Tom1 is described. Fifty-five assembled contigs, with an average length of 3698 nt, derived from TomDS, mapped with our plant virus database, representing six virus species previously found in the analysis of the total RNA sample (Table 2 and Table 3). The virus species not detected using dsRNA as the input was OLV1, although we found some mappings when reads were aligned against the OLV1 reference sequence (Table 3). For TomDS, the number of reads mapping to the identified reference virus sequences was substantially higher compared to the number of reads in Tom1 (32.16% versus 7.99%) (Table 2). This result was expected, as the dsRNA extraction method enriches preparations in virus-specific products, in this case in the replicative form of the ssRNA viruses [13]. Accordingly, the number of reads mapping to each viral genome, the average depth of sequencing and the percentage of the viral genome covered by the reads were similar or much higher for TomDS than for Tom1 (Table 3). The exceptions were TYLCV, for which the number of reads mapping to its genome and the average depth were slightly lower in TomDS (1344 and 69, respectively) than in Tom1 (1694 and 65, respectively), and RNAs 2, 3 and 4 from lettuce necrosis ringspot virus (LRNV), for which these numbers were significantly lower in TomDS than in Tom1 (Table 3). The nucleotide sequence identity between the viruses found in TomDS compared to Tom1 varied from 96.2% for PepMV to 99.9% for RNA4 from ToFBV (Table S1). In conclusion, the dsRNA-based method seemed to provide better enrichment in viral reads and the assembly of longer contigs than the total RNA-based method for RNA viruses, with some apparent exceptions.

### 3.4. In Silico Analysis of the Minimum Sequencing Depth Needed to Detect the Less Abundant Viruses

In an attempt to determine the minimum number of reads needed to detect the viruses infecting our tomato samples, we performed three different in silico simulations by decreasing the number of initial raw reads used in the bioinformatic analysis. For this, three subsets of raw data, consisting of 50% (Subset 1), 37.5% (Subset 2) and 25% (Subset 3) of the original Tom1 and TomDS reads were extracted randomly and analyzed following the same pipeline described previously (Table 4 and Table S3). We next measured three parameters in these datasets: number of mapped reads, percentage of coverage and average read depth along the viral genomes. 

**Figure 3:**
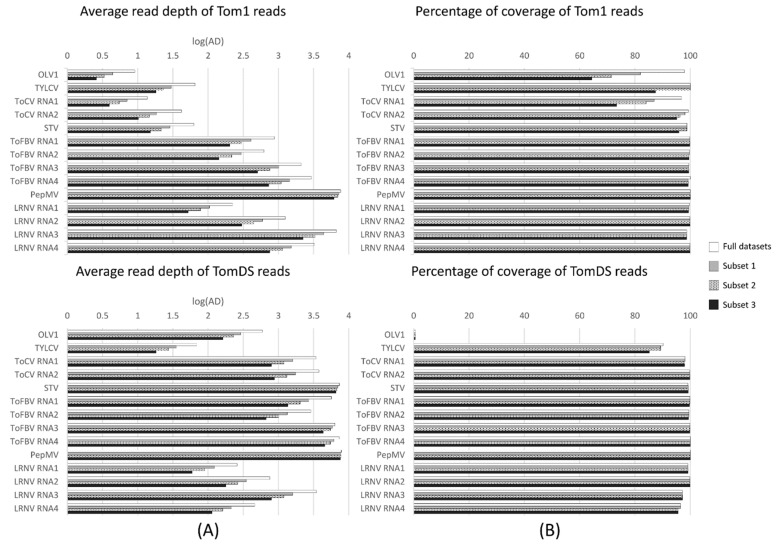
Comparison of average read depth and percentage of viral genomes covered by reads in Tom1 and TomDS. (**A**) Bar plots showing the logarithm of the average read depth for each viral genome in Tom1 (**upper**) and TomDS (**bottom**). (**B**) Bar plots showing the percentage of viral genomes covered by reads in Tom1 (**upper**) and TomDS (**bottom**). Full datasets: white bars; Subset 1: gray bars; Subset 2: dotted bars; Subset 3: black bars.

After the analysis of the different subsets (Table 4 and Table S3), we found the same viruses as when using the full datasets (seven species for Tom1 and six for TomDS), but there were differences in the estimated parameters. The percentage of unmapped reads against the tomato genome did not vary with the subsetting either for Tom1 or TomDS (Table 2 and Table 4). Although the number of contigs mapping to the plant virus database decreased with the subsets, the average contig length was not always lower, with the Subset 3 in Tom1 being higher (3167 nt) than for the full dataset in Tom1 (2788 nt). The number of reads mapping to the reference viruses decreased across the three subsets (Table 2 and Table 4); however, the percentage of the mapped reads was maintained among the three different subsets as compared to the full datasets (approximately 14.82% for Tom1 and 42% for TomDS) (Table 4). In general, the average read depth decreased across the different subsets, with the only exception being PepMV in TomDS, for which the average depth was maintained across the subsets (Figure 3 and Table S3). For the viruses in which the percentage of coverage using the full datasets was higher than 89%, there were only slight differences in the percentage of coverage when decreasing the sequencing depth (Figure 3 and Table S3). Four additional subsets, consisting of 12.50% (Subset 4), 6.25% (Subset 5), 3.13% (Subset 6) and 1.5% (Subset 7), were made (Table S3). For OLV1 in Tom1 and using 12.5% (Subset 4) of the total reads, 25 reads were mapped to the viral genome, with an average depth of 1.57 (Table S3), but no contigs longer than the minimum threshold could be assembled (Table S3). For TYLCV in TomDS, decreasing the raw reads to 3.13% (Subset 6) resulted in 38 reads mapping to its viral genome, with an average depth of 3.58 (Table S3), but again no contigs could be assembled. This in silico analysis was repeated using two new random subsets of 37.5% (Subset 2) and 25% (Subset 3) of the full datasets obtaining reproducible results (Table S3). In conclusion, to detect viruses infecting our sample pool, we could have decreased the initial sequencing depth to 25% of the full datasets for the total RNA input, from 80 M to 20 M, and to 6.25% for the dsRNA input, from 60 M to 3.75 M, and still identify the same virus species.

### 3.5. Viruses Already Reported to Infect Tomato Plants in Spain

Four viruses that are frequently reported in tomato crops were detected in our samples: PepMV, STV, ToCV and TYLCV. To identify their closest virus isolates in the databases, assembled contigs were further aligned by BLASTn against the specific virus taxon using the NCBI nr/nt database. We performed qRT-PCR or conventional RT-PCR to determine the presence of these viruses in the individual samples used for the pools (Table 1 and Table S2). PepMV (family Alphaflexiviridae, genus *Potexvirus*), a (+)ssRNA virus, was the most abundant virus in our sample pools, with more than 28 M reads across the three datasets: more than 13 M reads in both total RNA samples, and more than 15 M reads in the dsRNA sample (Table 3). Thirteen contigs, almost covering the complete genome with the typical genome features described for PepMV, were determined from the three samples, all of them mapping with the highest identity against both CH2 and EU strains. We detected PepMV using qRT-PCR in 19 out of 20 samples, 13 of them in mixed infections with viruses from both strains, and the other six infected only with viruses from the CH2 strain, which seemed to accumulate at higher concentrations in most of the samples (Table 1).

STV (family Amalgaviridae, genus *Amalgavirus*), which possesses a dsRNA genome, was the second virus for which a higher number of reads mapped along its genome in the dsRNA sample (more than 3 M), although very few viral reads were obtained from the total RNA samples (1782 in Tom1 and 1812 in Tom2) (Table 3). We were able to assemble almost the full viral genome from reads derived from the three datasets, obtaining three different contigs, one per dataset, that shared a percentage of identity higher than 99.9% among them (Table S1). These sequences contained the two overlapping ORFs described for STV: a putative CP and the RdRp protein. All the contigs showed a nucleotide identity of more than 99.9% with a sequence from Canada (MK610257.1). STV was detected in 16 out of 20 individual samples using conventional RT-PCR (Table 1).

ToCV (family Closteroviridae, genus *Crinivirus*), with a bipartite (+)ssRNA genome, was the fourth most abundant virus among the seven viruses identified, with 457,789 reads in TomDS and around hundred times less in the total RNA samples (3842 in Tom1 and 3870 in Tom2) (Table 3). A unique 8585-nt contig, covering almost the complete genome of RNA 1 from ToCV, was determined from the dsRNA sample. It shared 99.9% nucleotide identity with RNA 1 from the ToCV isolate from Spain (KJ200304.1). Multiple contigs were determined from Tom1 and Tom2 with a length shorter than 5777 nt, showing the highest identity with the same ToCV strain. Three contigs, ranging from 8195 nt to 8239 nt in length, that corresponded to the near complete genome of RNA 2 were determined from the three datasets respectively. These contigs showed more than 99.8% nt identity with RNA2 from the ToCV isolate from Spain (KJ200305.1). ToCV was detected in eight out of 20 individual samples by qRT-PCR (Table 1).

TYLCV (family Geminiviridae, genus *Begomovirus*), a (+) ssDNA virus, was detected using the two different RNA inputs but, with the exception of OLV1, it was the virus for which we obtained the lowest number of reads mapping to its genome (around 1694 and 1596 in Tom1 and Tom2 and 1344 in TomDS) (Table 3). Different contigs ranging from 685 nt to 2212 nt were found to derive from TYLCV across the three datasets. A BLASTn analysis of these sequences revealed a nucleotide similarity above 97% with other TYLCV sequences belonging to the Israel strain (TYLCV-IL). No insertion characterizing the TYLCV-IS76 isolates was detected in the non-coding intergenic region of these sequences [26]. Based on qRT-PCR of individual samples, TYLCV was detected in seven out of 20 samples.

### 3.6. Viruses Not Previously Reported to Infect Tomato Plants in Spain

We also identified three virus species that, to our knowledge, were not previously reported or not frequently reported to infect tomato plants in Spain: LRNV, OLV1 and ToFBV [27,28]. To further understand these observations, we determined the consensus sequences from the reads mapping to virus reference genomes. As for the other viruses, we detected the presence of these three viruses in the individual samples using qRT-PCR (Table 1 and Table S2).

LRNV (family Aspiviridae, genus *Ophiovirus*) is a four-segmented (−)ssRNA virus, and was detected in the pooled sample using the two RNA extraction approaches. We determined the consensus sequences for the four LRNV RNA segments (RNA 1 to 4). The RNA 1 consensus sequence (MW594439) was 7604 nt in length, covering 99% of the reference segment (AY535016), sharing a 99.4% identity with it. This RNA comprises two ORFs, which are 582 nt and 6834 nt in length, respectively. The RNA 2 consensus sequence (MW594440) was 1826 nt in length, lacking only 4 nt at the 5′ end compared to the reference RNA2 sequence (AY535017). Our sequence shared 99.2% at the nt level and 99.1% at the amino acid level with its RNA2 reference. The RNA 3 consensus sequence (MW594441) was 1505 nt (lacking 22 nt in total from both ends compared to the reference sequence, AY535018). Its vcRNA has one ORF of 1311 nt that encodes the CP protein. The CP showed a 99.1% amino acid identity with the CP of this reference isolate. The consensus sequence of RNA 4 (MW594442) was 1378 nt in length, covering 97% of the LRNV RNA 4 genome (AY535019) and sharing a 99.1% of nucleotide identity. The presence of LRNV was confirmed in the pooled sample as well as in four out of 20 individual samples by qRT-PCR using specific primers and probes targeting the CP (Table 1 and Table S2). The samples in which the virus was detected were from crops in Almería (Southeastern Spain) collected in 2015, 2019 and 2020, with the highest accumulation of this virus in samples collected in 2020 (Table 1), as determined by qRT-PCR. 

ToFBV (family Kitaviridae, genus *Blunervirus*) is a four-segmented (+)ssRNA virus, recently discovered to infect tomato plants in Italy and Australia [27]. The consensus sequences were determined for the four RNAs, and these were 5779 nt (RNA1, MW594435), 3586 nt (RNA2, MW594436), 2869 nt (RNA3, MW594437), and 1926 nt (RNA4, MW594438) in length, covering the full length of the four ToBFV RNA segments of isolate Fondi2018 from Italy (MK517477, MK517478, MK517479, and MK517480). The percentages of nucleotide identity of the consensus sequences against the reference sequences were 98.8%, 98.8%, 99.6% and 99% for RNAs 1 to 4, respectively. Five out of 20 individual samples were positive according to qRT-PCR using specific primers and probes targeting a conserved region of the RdRp (RNA1) (Table 1 and Table S2). Four samples were collected in Murcia in 2016, 2017 and 2019, and one in Portugal in 2015 (Table 1).

OLV1 (family Tombusviridae, genus *Alphanecrovirus*), with a (+)ssRNA genome, was the least abundant virus in our datasets (Table 3), and was only detected using the total RNA extraction method. Unlike the other two previous viruses, we compared contigs against the NCBI database to describe this virus, as the determined consensus sequence contained a very high proportion of unknown nucleotides. Four contigs, ranging from 641 nt to 1847 nt, were determined, covering a partial sequence of the RdRp protein and the full sequence of the CP. The contig covering the full CP gene shared 95.2% of nucleotide identity with isolate OLV1 Anhui from China (MK376952.1) and 98.1% amino acids with isolate A4P2 from Portugal (AHE40781.1). Two out of 20 samples were infected with OLV1 by qRT-PCR using specific primers and probes targeting the CP; both samples were collected in 2015 in Almería and Portugal, respectively (Table 1).

## 4. Discussion

HTS was used to identify viruses present in a pool of twenty samples of tomato leaves showing virus-like symptoms collected in areas where tomato cultivation is very important. We first assessed the reproducibility of the sequencing method by generating a replicate of the total RNA with a ribosomal depletion sample; after analysis, we did not find important differences between replicates in terms of virus species detected or the number of reads mapping to each reference virus genome. In contrast, our data suggest that the extraction method seems to have an impact on the viruses that could be identified by HTS, in agreement with previous reports. For instance, Kutnjak et al. [10] compared siRNA and VANA approaches and found that both provided highly similar viral mutational landscapes, but VANA allowed for better recovery of complete viral genomes and detection of recombinant genomes [10]. In another example, rRNA-depleted total RNA was found to be superior to siRNA for the identification of citrus tristeza virus (CTV) (family Closteroviridae, genus *Closterovirus*) and citrus dwarfing viroid (CDVd) (family Pospiviroidae, genus *Apscaviroid*) infecting grapefruit, rendering better coverage for CTV but not for the viroid [29]. Another study showed that the performance of these two approaches tended to be virus-dependent, but in general longer contigs and higher genome coverage were obtained by rRNA-depleted total RNA than by sRNA sequencing [11]. Ma et al. [12] compared dsRNA and VANA approaches in assessing the virus diversity in wild plant populations. In their experimental system, the dsRNA approach revealed a broader and more comprehensive diversity for RNA viruses than VANA [12]. Gallo-García et al. [30] used total RNA and dsRNA as inputs to assess the virus populations in cape gooseberry (*Physalis peruviana* L.). They found higher sequence diversity for a specific virus species in total RNA as compared to dsRNA, but the total RNA extraction method failed to detect viruses present at low concentrations [30]. Hence, these and other authors have suggested the use of rRNA-depleted total RNA and dsRNA as complementary methods to obtain a comprehensive picture of the viruses present in a field sample [3,30]. In our case, and contrary to what was described by Gallo-Garcia et al. [30], rRNA-depleted total RNA performed better because seven, as opposed to six, viral species were detected using rRNA-depleted total RNA versus dsRNA. However, we cannot rule out the possibility that this difference may be due to the different pooling strategies used here. Although there were reads mapping to OLV1 in the dsRNA sample, the percentage of read coverage along the viral genome was only 0.62% (Table 3). This coverage was rendered by a fragment of 22 nt inside a read of around 109 nt that had been sequenced many times, hence the high average depth found (Table 3). However, this read had no significant hits against the NCBI database, not even with OLV1. Both methods allowed almost complete coverage of the genomes of the most abundant viruses (PepMV, STV, LRNV, ToFBV), however, the average depth was higher using the dsRNA approach in most of the cases. Interestingly, we noticed that total RNA generated more reads for LRNV, a (−)ssRNA virus, and for TYLCV, a ssDNA virus. This latter result was not surprising, as during TYLCV replication no dsRNA replicative intermediates are formed [31].

Some previous works have discussed the possibility of the application of HTS for routine plant virus diagnostics, mentioning different parameters to take into consideration, such as sensitivity, specificity and reproducibility [32]. Al Rwahnih et al. [33] compared the sensitivity of HTS to biological indexing for plant material certification in grapevines and concluded that it may reach a high sensitivity level, with the advantage of being time effective as compared to conventional methods [33]. In addition, Candresse et al. [34] used HTS to detect sugarcane white streak virus (family Geminiviridae, genus *Mastervirus*) in two quarantined sugarcane plants, showing the importance of including this method to assess plant health status [34]. However, sequencing large numbers of individuals or samples, which is often needed to obtain an overview of the plant viruses present in a population, is still challenging, despite falling HTS costs over the last decade. Furthermore, the need for a high-quality RNA input for library preparation and the complexity of the bioinformatics analyses should be taken into consideration when approaching HTS studies. One of the main methodological decisions for pool sequencing is the balance between the number of samples to be pooled and the sequencing depth required to detect all viruses present in the pool. According to the literature, the pool composition of different metagenomic studies for plant viromes varied from four to 50 individual samples per pool [9,12,35,36,37,38,39]. Skums et al. [40] provided a mathematical approach for a pooling strategy for the massive sequencing of human viruses, since the complex nature of these samples imposed restrictions on the pool design [40]. Here, we tried to address the problem of minimum reads needed to detect the less abundant viruses in a pool by following an in silico approach. The libraries for the two strategies used were sequenced with a different initial sequencing depth: 80 M for the total RNA replicates and 60 M for the dsRNA sample. In theory, this sequencing depth would correspond to 4 M reads per sample for the total RNA and 3 M reads per sample for the dsRNA input. We have demonstrated that we were able to detect the same viruses from the full datasets and from the three different subsets composed of 50%, 37.5% and 25% of the initial reads, and even for two additional subsets composed of 12.5% and 6.25% of the initial reads in the case of dsRNA. The less abundant viruses identified in the full datasets were OLV1 in the case of the total RNA replicates and TYLCV in the case of the dsRNA sample. However, the percentage of coverage decreased in the subsets, so the level of confidence in the detection of these viruses also decreased. TYLCV and OLV1 were detected in seven and two individual samples, respectively, and at very high Ct values (higher than 28 in most of the samples), suggesting the low accumulation of these viral RNAs in the pooled sample. In conclusion, we believe that 1.5 M reads per sample could have been used for assessing the tomato virome when using rRNA-depleted total RNA. In the case of using dsRNA as the input, a minimum of 0.2 M reads per sample could have been used as the initial sequencing depth. Generalizing these results is difficult, as different crops under different environmental conditions infected by different sets of viruses may require other sequencing depths. Using a low number of reads in a de novo assembly, it is possible that bioinformatics analysis fails to build significantly long contigs; hence, some virus derived reads could be disregarded during the bioinformatic analysis. The high output noise generated by this technique and/or possible contaminations demonstrate the necessity of using conventional detection methods as a complementary tool for the confirmation of the presence of a virus.

Three viruses known to infect tomato plants, and to induce important crop losses, were detected in our samples. PepMV was detected in 19 samples by means of qRT-PCR, confirming the HTS results. Two strains, EU and CH2, were detected in mixed infections in 13 out of 19 PepMV-infected samples. This high incidence could be due to the generalization of cross-protection as a means of disease control in the South of Spain [41], though generalized single and mixed infections of PepMV isolates of these two strains were already reported in the region before the extended use of cross-protection [42]. The detection here of ToCV and TYLCV is not surprising as both are prevalent viruses across the southern and eastern regions of Spain and both are transmitted by whiteflies [43,44,45,46]. A survey of STV incidence was conducted on different tomato fields in Spain in 2018, revealing that STV was widespread [47]. Moreover, this virus was detected in different tomato varieties and nurseries, but STV-infected tomato plants did not show any disease symptoms [47]. Apart from the four viruses mentioned above, there are other viruses known to be widespread and of major concern for tomato plants, including, for instance, TSWV, potato virus Y, cucumber mosaic virus [1] and the emergent ToBRFV, but none of these were detected in our samples.

In addition to viruses frequently cited in tomato plants, three viruses that are seldom if ever cited to infect these species were detected in this study. The first report of LRNV was in lettuce (*Lactuca sativa*) crops in 1996, associated with lettuce ring necrosis disease. LRNV is transmitted by the soil-borne fungus *Olpidium brassicae* [48]. However, no additional information on the distribution and the epidemiological status of the virus could be found in the literature. Although in our study LRNV was detected in samples showing necrosis, vein clearing and yellow mosaic, these symptoms can be hardly associated with it, because it was detected in mixed infections in all the positive samples. OLV1 was isolated for the first time from olive trees in the Apulia region of Southern Italy [49]. Infected olive trees had normal flowers and fruits and did not show disease symptoms except for occasional fasciation and bifurcations of leaves and twigs [49]. Since then, OLV1 has been reported in different hosts in various countries [50,51], and in 2010 it was reported for the first time in tomato plants in Poland, associated with necrotic spots on the leaves [28]. The presence of this virus in the plants of different tomato cultivars was restricted to local lesions or to necrotic areas [28]. This may suggest that the surrounding crops may constitute the primary natural source of virus inoculum in the greenhouse-grown tomato, and that the daily manipulation of tomato plants by workers could play a key role in its spread. ToFBV is a new blunervirus that was recently reported in Italy and Australia, and it was associated with blotchy ripening and dimpling of the tomato fruits [27]. ToFBV could not be transmitted mechanically to either tomato or a set of various herbaceous plants, and thus Koch’s postulates have not been fulfilled yet for this virus. Generally, kitaviruses share important epidemiological aspects such as symptomatology, lack of systemic movement and mite-mediated transmission [52].

The analysis of the individual samples, carried out to detect the seven viruses, revealed the extent of mixed infections, and the almost universal mixed infections of any of the viruses with CH2 and/or EU isolates of PepMV. Multiple viruses could infect a single plant; for instance, LRNV was detected in plants infected with OLV1, TYLCV and ToCV. ToFBV was detected in mixed infections with TYLCV and ToCV. Mixed infections can affect the virus’s replication and movement competence, transmission capacity, virulence, host range and symptom severity [53]; therefore, more studies must be conducted to assess the impact of mixed infections in tomato and other crops. Mixed infections also prevented us from associating virus detection with disease symptoms—using the data collected during our sampling, no obvious correlation could be established between observed symptoms and the detection of any of the three viruses discussed above.

HTS is a very powerful technique for virus discovery and detection, and this emerged clearly from our study. We identified viruses that were present in tomato crops several years ago (samplings were conducted in 2015) but which remained unreported. The increased ability to detect new or infrequent viruses using this technique raises several questions relating to how to deal with them from a crop protection point of view, as well as the complexity of their biological characterization, particularly for the newly-identified plant viruses and viroids, and their impact at biosecurity, commercial, regulatory and scientific levels [54]. Here, we have reported for the first time the presence of OLV1 in tomato crops in Portugal and the South of Spain, and the presence of LRNV and ToFBV in tomato crops in the South of Spain. However, broader surveys are needed to assess the prevalence and potential impact of these viruses, including surveys of alternative hosts that may serve as virus reservoirs, in order to better understand their epidemiological status.

## Figures and Tables

**Table 1:** Description of samples and confirmation of viral infections by means of qRT-PCR or conventional RT-PCR.

Sample ID	Surveyed	Location	Symptoms	Virus Detected ^1^							
				OLV1	TYLCV	ToCV	STV ^2^	ToFBV	PepMV-EU	PepMV-CH2	LRNV
R20-01	March 2020	Almería	Vein clearing	−	−	−	+	−	++	+++	+++
R19-12	November 2019	Almería	Necrotic spots on leaves	−	−	+	+	−	+	+++	+
R19-09	October 2019	Almería	Leaf curling, leaf mosaics	−	−	−	+	−	+++	+++	−
R19-08	October 2019	Almería	Leaf curling, leaf mosaics	−	−	−	−	−	+++	++	−
R19-07	September 2019	Almería	Chlorosis, yellow spots on leaves, leaf mosaics	−	−	−	+	++	+++	++	−
R17-01	Febuary 2017	Murcia	Upward curling of leaves, chlorosis on leaves	−	−	−	−	++	+	++	−
H-57	December 2016	Murcia	Leaf mosaics	−	−	−	+	−	++	++	−
H-55	June 2016	Murcia	Leaf distortion	−	−	+	+	−	−	++	−
H-54	May 2016	Murcia	Leaf distortion	−	−	−	+	−	++	++	−
H-53	May 2016	Murcia	Leaf distortion	−	+	+	+	+++	+	+++	−
H-52	May 2016	Murcia	Distortion and mosaic in fruit	−	+	+	+	+++	−	+++	−
H-50	April 2016	Murcia	Leaf distortion	−	−	−	+	−	+	+++	−
H-43	December 2015	Granada	No clear symptoms	−	−	−	+	−	−	+	−
H-42	December 2015	Granada	Leaf curling	−	++	−	−	−	+	+	−
H-31	October 2015	Almería	Yellow mosaic	−	++	+	+	−	−	−	−
H-20	April 2015	Portugal ^3^	No clear symptoms	−	−	+	+	++	−	+++	−
H-13	Aprli 2015	Portugal ^3^	No clear symptoms	−	+	−	−	−	−	+++	−
H-11	April 2015	Portugal ^3^	No clear symptoms	++	−	−	+	−	−	++	−
H-10	April 2015	Almería	Necrosis, yellow mosaic and distortion of leaves	−	+	++	+	−	+	++	+
H-09	April 2015	Almería	Necrosis, yellow mosaic and distortion of leaves	+	+	++	+	-	+	+++	+

^1^ Relative amount of viral RNA denoted as follows: +++ 14 < Ct < 18; ++ 18 < Ct < 28; + Ct > 28; Ct: cycle threshold; ^2^ conventional RT-PCR; ^3^ Torres Vedras (Lisbon).

**Table 2:** Summary of sequencing and mapping results.

	Tom1		Tom2		TomDS	
	Reads	%	Reads	%	Reads	%
Raw reads	86,284,538		84,739,174		64,540,826	
Clean reads	85,026,574	98.54	83,516,356	98.56	63,715,596	98.72
Host mappings	40,905,042	48.11	39,992,206	47.89	16,395,174	25.73
Filter reads	44,121,532	51.89	43,524,150	52.11	47,320,422	74.27
Viral contigs	63		51		55	
Unique viruses	7		7		6	
Viral reads	6,790,296	7.99	7,159,776	8.57	20,491,882	32.16

**Table 3:** Summary of mapping of reads against identified viral genomes.

Virus	Accession	Genome	Segment	Ref. Length	Tom1			Tom2			TomDS		
					Reads	AD	PC	Reads	AD	PC	Reads	AD	PC
OLV1	DQ083996	(+)ssRNA		3702	390	9	97.97	364	9	89.68	678	590	0.62
TYLCV	HF548826	(+)ssDNA		2787	1694	65	100	1596	60	99.64	1344	69	90.17
ToCV	KF018280	(+)ssRNA	RNA1	8596	1106	14	96.92	1076	15	94.16	222,112	3441	98.15
	KJ815045		RNA2	8249	2736	42	99.33	2794	43	99.52	235,672	3759	99.79
STV	KT438549	dsRNA		3463	1782	63	98.84	1812	64	98.64	3,459,440	7319	99.19
ToFBV	MK517477	(+)ssRNA	RNA1	5811	39,452	878	99.78	41,498	930	99.78	255,152	5665	99.88
	MK517478		RNA2	3643	17,810	626	99.75	17,760	628	99.45	79,414	2892	99.56
	MK517479		RNA3	2872	72,830	2096	99.51	81,684	2417	99.65	500,460	6360	99.93
	MK517480		RNA4	1946	47,102	2938	100	51,060	3158	100	317,660	7309	100
PepMV	NC_004067	(+)ssRNA		6450	6,431,722	7687	100	6,809,266	7686	100	15,351,220	7831	100
LRNV	NC_006051	(−)ssRNA	RNA 1	7651	13,116	223	99.76	10,716	183	99.48	14,738	258	99.12
	NC_006052		RNA 2	1830	17,546	1258	99.89	15,668	1124	99.95	10,512	758	99.89
	NC_006053		RNA 3	1527	108,412	6655	98.76	95,402	6507	99.41	38,700	3458	97.12
	NC_006054		RNA 4	1417	34,598	3226	99.86	29,080	2749	98.52	4780	462	96.47

AD: average read depth; PC: percentage of reference sequence covered by reads.

**Table 4:** Summary of results obtained after subsetting the raw reads.

	Subset 1 (50%)				Subset 2 (37.5%)				Subset 3 (25%)			
	Tom1		TomDS		Tom1		TomDS		Tom1		TomDS	
	Reads	%	Reads	%	Reads	%	Reads	%	Reads	%	Reads	%
Subset	40,000,000		30,000,000		30,000,000		22,500,000		20,000,000		15,000,000	
Clean reads	39,416,281	98.54	29,616,416	98.72	29,562,358	98.54	22,212,742	98.72	19,708,041	98.54	14,809,024	98.73
Host mappings	19,544,446	49.58	7,618,386	25.72	14,223,278	48.11	5,714,092	25.72	9,483,289	48.12	3,808,670	25.72
Filter reads	20,455,554	51.90	21,998,030	74.28	15,339,080	51.89	16,498,650	74.28	10,224,752	51.88	11,000,354	74.28
Viral contigs	50		56		43		40		36		40	
Unique viruses	7		6		7		6		7		6	
Viral reads	3,033,136	14.83	9,245,404	42.03	2,275,725	14.84	6,934,690	42.03	1,515,690	14.82	4,622,820	42.02

## Data Availability

Virus sequences were deposited in the GenBank database under the following accession number: ToFBV (MW594435, MW594436, MW594437 and MW594438 for RNA1, RNA2, RNA3 and RNA respectively and LRNV (MW594439, MW594440, MW594441 and MW594442 for RNA1, RNA2, RNA3 and RNA4 respectively). Raw reads were submitted to the Sequence Read Archive (SRA) under the ID numbers SRR14066946-SRR14066948. Main code was deposited in GitHub (https://github.com/ldonaire/R_scripts/blob/main/Tomato_virus_pipeline_-v1.R; accessed on May 2020).

## Supplementary Materials

The following are available online at https://www.mdpi.com/article/10.3390/microorganisms9051043/s1, Table S1. Comparison of percentage of identity of virus sequences among the three samples; Table S2. Primers and probes used for viral detection by qRT-PCR and conventional RT-PCR for STV; Table S3. Summary of results obtained after mapping the reads from different subsets against identified viral genomes.
